# Psychosis as a Manifestation of Focal Impaired Awareness Seizure in a Geriatric Patient

**DOI:** 10.7759/cureus.41838

**Published:** 2023-07-13

**Authors:** Oluwaseun Oke, Bamidele O Johnson, Shahzad Chida, Victor Kekere, Shiraz Azim

**Affiliations:** 1 Psychiatry and Behavioral Sciences, Interfaith Medical Center, Brooklyn, USA

**Keywords:** geriatric, aura, hallucinations, delusions, psychosis, temporal lobe, focal impaired awareness seizure

## Abstract

Focal impaired awareness seizures (FIAS), previously known as complex partial seizures, refer to focal seizures that start in one hemisphere of the brain and are associated with an impairment in consciousness. FIAS of temporal lobe origin most often present with psychopathology, such as behavioral and affective symptoms. It has a bimodal age distribution peaking at the extremes of life. Geriatric presentations can often be subtle and go unnoticed asides from a few symptoms, such as confusion and memory lapses. Here, we present a unique case of a geriatric patient with FIAS presenting as transient psychosis.

## Introduction

Focal impaired awareness seizures (FIAS), previously known as complex partial seizures, refer to focal seizures that start in one hemisphere of the brain and are associated with an impairment in consciousness [1]. They are also sometimes referred to as temporal lobe seizures or psychomotor seizures. FIAS arises from the temporal lobe in 60% of cases or the frontal lobe in 30% of cases [1]. In geriatric patients, FIAS accounts for 40% of all seizure types; however, the proportion with temporal lobe seizure is uncertain [2]. Aura, altered consciousness, and automatisms characterize FIAS. Among all seizures, FIAS of temporal origin most often presents with psychopathology [3,4]. The incidence rate of seizures ranges from 34.7 to 54.3 per 100,000 population. Temporal lobe seizure accounts for 10.4 per 100,000 population and is the most common type of partial seizure [5]. The overall prevalence of psychiatric disturbances in patients with seizures is estimated to be between 20% and 30% [6]. Due to its affective, behavioral, and cognitive symptoms, FIAS is often misdiagnosed as a primary psychiatric disorder. Usually, these symptoms are present with others, which are atypical for diagnosing primary psychiatric illness, such as macropsia, micropsia, gustatory and olfactory hallucinations, intense, short-lived delusion, and déjà vu phenomenon [7].

FIAS presents as aura followed by ictal, postictal, and inter-ictal states. Auras may be classified by symptom type, including cognitive or psychic, emotional or affective, and sensory or autonomic symptoms [8]. Cognitive or psychic symptoms include déjà vu, dissociation, depersonalization or derealization, forced thinking, and aphasia or dysphasia. Emotional or affective symptoms include agitation, aggression, anger, anxiety, fear, paranoia, pleasure, crying, or laughing. Sensory symptoms include auditory, gustatory, hot-cold sensations, olfactory, somatosensory, vestibular, and visual [8]. Autonomic symptoms include heart rate changes, flushing, gastrointestinal, pallor, piloerection, and respiratory [8,9]. Chronic psychosis following seizures (symptoms greater than three months) is often difficult to distinguish from primary psychotic illness but is associated with preservation of effect and lack of negative symptoms [10].

In geriatric patients, the diagnosis of FIAS poses a challenge. The patients can be unaware of seizures due to the subtle nature of the clinical features. It may sometimes only present as confusion, brief gaps in the flow of conversation, memory lapses, and blank stares. In the younger age group, identifiable associations often include brain tumors, CNS infections, and head trauma, while geriatric presentations are often associated with a cerebrovascular accident. However, up to 50% of the elderly can go without an identifiable cause [2]. This case report presents a unique case of a geriatric patient with FIAS presenting as transient psychosis.

This case report was previously presented as an abstract at the 2023 American Psychiatric Association Annual Meeting on May 20, 2023.

## Case presentation

Ms. K is a 67-year-old African American female with no past psychiatric history and a past medical diagnosis of hypertension and chronic kidney disease. She presented to the emergency department with an abrupt onset of disorganized behavior. The patient was seen running on the street without clothes, yelling, and was brought to the hospital via EMS. She presented in her underwear to the medical emergency department, refused to provide identifying information, and did not cooperate with medical personnel. She was given emergency medication for psychotic agitation. On initial psychiatric evaluation, she was uncooperative and exhibited mutism and staring. She was suspected to be catatonic and was given intramuscular lorazepam.

On psychiatric re-evaluation 12 hours after the initial presentation, the patient was calm and fully cooperative. The patient reported that prior to the hospital presentation, she experienced command auditory hallucinations of Jesus instructing her to sing, take off her clothes, and walk outside. She reported that the voice was only meant for her. She denied seeing Jesus at the time. At the time of re-evaluation, the patient denied any psychotic, mood, or anxiety symptoms.

On mental status examination, she was alert and oriented in time, place, and person. Her thought process was linear, logical, and goal-directed. Her thought content was devoid of delusions, suicidal/homicidal ideations, intent, or plan, and she did not endorse any perceptual disturbance.

Per collateral information, the patient had presented at another hospital two days prior because of irrational behavior, agitation, and combativeness. Per a hospital chart review, the patient had reported that before the onset of symptoms, she went into a room with a "really weird smell." She reported that after that, she felt strange. The patient was discharged from the previous hospital following overnight observation, intravenous rehydration, management of psychotic agitation, negative medical workup, and psychiatric clearance.

After medical evaluation in the emergency department, the patient was admitted to the medical unit for the management of acute kidney injury on chronic kidney disease secondary to dehydration, and for further evaluation of intermittent behavioral changes. She was found to have elevated creatinine levels, which had increased from baseline values. There were no uremic symptoms observed. A neurological evaluation by the neurology team was placed to identify the underlying cause of the intermittent behavioral changes and fall. Based on the evaluation, there was a high index of suspicion of FIAS.

Investigations

Complete blood count and urinalysis were within normal limits. Urine toxicology was positive for benzodiazepine (the patient had earlier received midazolam and lorazepam for psychotic agitation and catatonia, respectively). The complete metabolic profile showed no electrolyte derangement, but creatinine was found to be elevated (3.49 mg/dl) from previous baseline values (2.98 mg/dl).

**Figure 1:**
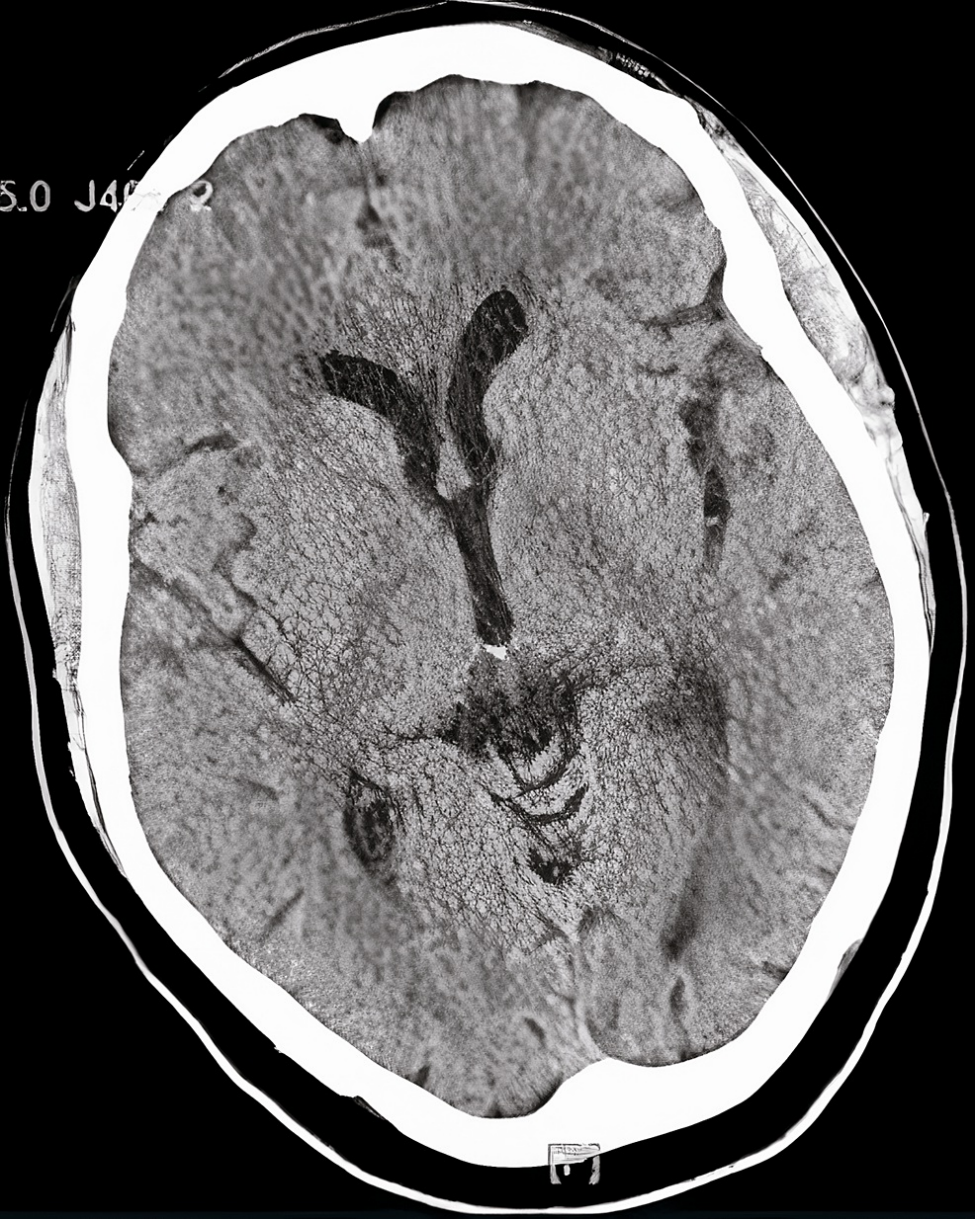
CT scan of the brain showing left temporal lobe volume loss.

Computed tomography (CT) scan showed left temporal lobe volume loss (Figure 1). Electroencephalogram (EEG) and magnetic resonance imaging (MRI) were scheduled to be completed during outpatient follow-up; however, there was no report available during the chart review.

Differential diagnoses

The presentation of altered mental status and findings of mild dehydration suggested a possibility of delirium. However, this was ruled out after evaluation by the neurology team. The only medical evidence of possible dehydration was determined not significant to result in an altered mental state in this patient. The patient had also presented 48 hours earlier at another emergency room where she was rehydrated with intravenous fluids. There was no other significant identifiable evidence from the patient's history, physical examination, and laboratory findings that the acute change in mental status is a direct physiological consequence of another medical condition, substance intoxication or withdrawal, or exposure to a toxin. Her urine toxicology was negative for all substances except for therapeutic benzodiazepine administered in the hospital.

Another differential diagnosis was dementia with behavioral changes. The neurological team's evaluation also ruled this out. Based on the patient's history, collateral information, and physical examination, this presentation was acute in onset, and there was no indication of a significant decline in cognitive function that interfered with her everyday activities.

Treatment

On presentation in the medical ED, the patient was managed for psychotic agitation with intramuscular haloperidol 5 mg and intramuscular midazolam 4 mg. The patient was also given intravenous lorazepam for suspected catatonia.

Intravenous fluid normal saline 0.9% was given to manage the patient's acute kidney injury secondary to dehydration. Oral valproic acid 250 mg daily was given to manage the seizure disorder.

Outcome and follow up

The patient was discharged on day three. Valproic acid 250 mg daily was continued in the outpatient, and at the one-month follow-up, the patient reported no further symptoms of psychosis, behavioral changes, or seizures.

## Discussion

Patients with FIAS may manifest a wide variety of clinical features, including affective, behavioral, and cognitive symptoms [11]. Associated delusions and hallucinations can be of religious themes [4,12]. As a result, patients presenting with this condition can often be misdiagnosed as having a psychiatric disorder. Ms. K presented 48 hours apart in two different hospitals with similar psychotic presentations, and both episodes resulted in the administration of haloperidol and lorazepam. She reported no further episodes after the second hospitalization following a prescription for anti-seizure medication.

Ms. K typifies a patient who can be easily misdiagnosed, as she is elderly and presented with perceptual, behavioral, and cognitive disturbances. In this case, the behavioral and cognitive components were more predominant. Her presentation particularly is seen with ictal psychosis. They are often in connection with typical temporal lobe automatisms. The presence of automatisms and other typical epileptic phenomena can help the clinician distinguish a nonconvulsive status with psychic symptoms from a brief psychotic episode [13]. The automatisms often involve the hands (fumbling, picking, fidgeting) or mouth (chewing, lip smacking, swallowing). Less common include vocalizations, ictal speech, and affective behaviors (out-of-context fear). Additionally, even less common behaviors, such as crying (dacrystic), laughing (gelastic), and so-called "leaving behaviors," for example, running out of the house or down the street during a seizure (cursive), have been reported [13]. A rare automatism, whistling, has also been recently reported to occur during temporal lobe seizures [14]. Aura often characterizes FIAS and may present as recurrent intrusive thoughts, hallucinations of unpleasant smells, fear, and tachycardia.

Often electroencephalogram (EEG) can be used to diagnose seizure disorder, but findings can be non-specific in FIAS, thus requiring further workup. When EEG findings are available, they can be diagnostic and often show spike or sharp waves in the tip or front of the temporal lobe [15]. Some research has shown evidence of temporal lobe atrophy in CT scans in patients with temporal lobe epilepsy. Mesial temporal lobe sclerosis has been seen mainly in elderly persons with new-onset seizures [2]. Neuroimaging, particularly MRI, can be a sensitive and specific imaging technique for localization-related epilepsy. This can detect subtle abnormalities, which may serve as an area of the seizure focus [16].

## Conclusions

This case highlights the importance of thorough clinical evaluation, especially in geriatric patients with a presentation of psychosis, altered mental sensorium, and associated chronic medical conditions. A high index of suspicion is needed for patients with atypical presentations of seizure disorders to properly manage and prevent future seizure episodes, particularly in the absence of a definitive diagnostic tool at the time of seizure activity. Clinicians should be aware that seemingly psychiatric presentations in geriatric patients may be manifestations of underlying seizure disorders.
